# Impact of second forward-view examination on adenoma detection rate during unsedated colonoscopy: a randomized controlled trial

**DOI:** 10.1186/s12876-021-01783-9

**Published:** 2021-05-10

**Authors:** Keshu Shan, Hongpeng Lu, Zhixin Zhang, Jiarong Xie, Lu Xu, Weihong Wang, Chunjiu Hu, Lei Xu

**Affiliations:** 1 China Department of Gastroenterology, Ningbo First Hospital, 59 Liuting St, Ningbo, 315010 Zhejiang China; 2 College of Medicine, Ningbo University, Ningbo, 315010 Zhejiang China

**Keywords:** Second forward-view examination, Adenoma detection rate, Polyp detection rate, Colonic polyps, Colorectal cancer, Colonoscopy

## Abstract

**Objectives:**

Colorectal cancer on the right side of the colon has been suggested to be harder to detect by colonoscopy. The aim of this study was to evaluate whether a second forward-view examination of the right side of the colon could increase the adenoma detection rate (ADR) and/or polyp detection rate (PDR).

**Methods:**

This was a single-centre randomized controlled trial. Patients undergoing colonoscopy were recruited and randomly assigned to the second forward-view examination (SFE) group, in which the right side of the colon was examined twice or the traditional colonoscopy (TC) group in which the colonoscopy was performed in a standard manner. The primary outcome was the ADR of right colon. The overall PDR and ADR, PDR of the right colon, per-adenoma miss rate of the right colon, and advanced lesion detection rate were also recorded and compared.

**Results:**

A total of 392 patients were included in the study (SFE group 197 vs. TC group 195). The ADR and PDR of the right colon in the SFE group were significantly higher than those in the TC group (ADR 10.7% vs. 5.1%; *P* = 0.042); PDR 17.8% vs. 9.7%, *P* = 0.021). No significant difference was found in overall PDR/ADR, or advanced lesion detection rate between the two groups.

**Conclusions:**

This prospective controlled study revealed that a second forward-view examination could modestly increase the ADR and PDR of the right colon during unsedated colonoscopies. This simple, safe and time-effective technique might be recommended for routine unsedated colonoscopy.

*Trial registration:* Clinical Trials.gov, NCT03619122. Registered on 7/8/2018.

**Supplementary Information:**

The online version contains supplementary material available at 10.1186/s12876-021-01783-9.

## Introduction

Colorectal cancer (CRC) is one of the most commonly diagnosed malignant neoplasms worldwide [1], the incidence of which has increased in recent decades [2]. Colonoscopies and endoscopic polypectomy have been indicated to be an effective way to prevent CRC and decrease the mortality of CRC [3]. Thus, the adenoma detection rate (ADR) is widely accepted as an important quality indicator for colonoscopy [4]. However, many studies [5, 6] have reported that interval CRCs that are detected after a prior colonoscopy still account for 0.6% to 9% of colorectal cancers. Furthermore, several studies [7, 8] demonstrated that colonoscopy provides lower detection rate of CRCs in the proximal colon than in the distal colon, which means that interval CRCs are more likely to develop proximally than distally. One of the plausible theories is that polyps are easily missed due to the proximal aspect of the folds. Additionally, flatter lesions located in the proximal colon tend to be sessile serrated adenomas which are regarded as probable precursors of CRC [9]. Therefore, it is critical to develop a practical manipulation to improve the adenoma/polyp detection rate of the right colon.

A number of techniques have been implemented to achieve a potential improvement in the detection of adenomas in the proximal colon, such as repeated examination, cap-assisted colonoscopy [10], and use of a third-eye retroscope [11]. Among these methods, a second forward-view examination of the proximal colon may be the easiest and most convenient method for endoscopists to perform, as no additional equipment, staff or expenses are required. A prospective cohort study of 280 patients revealed additional adenomas in 15.4% of patients with an increase in the ADR in the right side of the colon by 6.7% [12]. Another prospective trial with 400 patients found that the increase in the ADR was 2.3% when a repeated forward-view examination was performed [13]. These back-to-back trials were all performed by two different endoscopists, which might be unrealistic in the real world. Although retroflexion is reported to have a high success rate in terms of maneuverability and a lower risk of adverse events [14–16], endoscopists are more likely to perform forward-view examinations in routine clinical practice. We aimed to evaluate the effect of forward-view examinations of the right colon on ADR performed by one endoscopist.

Nevertheless, colonoscopy has known to cause pain and discomfort among the general public. Sedated colonoscopies are routinely performed in the USA and Western countries [17]. To the best of our knowledge, almost all the relevant re-examination studies published to date have been conducted under sedation. However, no study has evaluated the influence of including only unsedated patients on adenoma detection by means of re-examination during colonoscopy.

We therefore conducted a prospective randomized-controlled trial to evaluate the impact of second forward-view examination of the right colon on ADR and PDR in patients undergoing colonoscopies without sedation.

## Methods

### Study design

A single-centre, randomized controlled trial was performed in the Endoscopy Department of Ningbo First Hospital from September 2018 to June 2019. The study protocol was approved by the Ethics Committee of Ningbo First Hospital (2018-R014) and was registered on ClinicalTrials.gov (ID: NCT03619122) on 7/8/2018. The trial complied with the Declaration of Helsinki and written informed consent was obtained from all participants prior to inclusion. Moreover, standardized manual bowel preparation was performed for all participants. Patients who underwent colonoscopy were randomly assigned to the traditional colonoscopy (TC) group or second forward-view examination (SFE) group.

### Participants

Outpatients (18–75 years old) who were scheduled to undergo colonoscopy for screening or surveillance at Ningbo First Hospital from September 2018 to June 2019 were recruited. Patients were excluded if they had a history of colon resection, inflammatory bowel disease or polyposis syndromes, or poor bowel preparation (Boston Bowel Preparation Scale [BBPS] score < 2 in any segment of the colon) [18]. Participants who were unable to provide informed consent, did not successfully undergo caecal intubation, or were receiving active antithrombotic therapy preventing polypectomy were also excluded.

### Randomization and colonoscopy procedures

The computer-generated randomization numbers were sealed in an envelope. Included participants were randomly assigned to one of the two groups: the second forward-view examination (SFE) group, in which the right colon was examined twice in forward view; or the traditional colonoscopy (TC) group, in which a standardized colonoscopy procedure was performed. Complete caecal intubation was defined when the ileocecal valve and appendicular orifice were seen.

All participants received a single 3 L dose of polyethylene glycol (PEG) 5–6 h before the scheduled examination time. An educational video of bowel preparation was sent to all of patients via mobile phone. Baseline demographic characteristics including age, sex, weight, height, previous history of surgery, family history of colorectal cancer, etc. were recorded by one of the assistants prior to colonoscopy. All colonoscopies were conducted by one of four gastroenterological endoscopists who perform approximately 500–800 colonoscopies annually (Additional file 1). High-definition colonoscopes (Olympus CF-HQ290I/CF-H290I/CF-HQ290ZI, Japan) were used for all procedures.

All colonoscopies were performed without anaesthesia. We conducted an initiation meeting before the launch of the clinical study, requiring every doctor to perform a routine insertion method, and in a fixed withdrawing technique, which was the spiral back technique. In addition, a timer was used to adjust withdrawal time. After successful insertion into the caecum, the scope was withdrawn to the hepatic flexure allowing the colonic mucosa to be carefully examined. At this moment, the sealed envelope with a random number was opened. If the patient was allocated to the TC group, the scope was directly withdrawn to the anus. If the patient was assigned to the SFE group, the colonoscope was advanced to the caecum again for a second inspection of the right side of the colon, and then passed to the anus. The withdrawal time was required to be at least 6 min. The time for the second examination was not included in the withdrawal time. Whether the patient’s position was shifted during the procedure was decided by the operators.

For all endoscopies, caecal intubation time, withdrawal time, and second examination time were documented by assistants during the procedure, exclusive of therapeutic time. The adequacy of bowel preparation was scaled according to the BBPS by the endoscopists. The number, size, location, and morphology of polyps were also recorded. Endoscopic polypectomy or biopsy was performed when necessary. Only if the participants refused polypectomy and the operators’ suggestion failed, was biopsy rather than polypectomy performed. The samples were submitted for pathological assessment. The size of the polyp was measured by visual comparison with opened forceps or a snare.

### Sample size

The sample size was calculated on the basis of a previous study. We set the ADR of the right colon in the traditional group at 15%, and the ADR of the right colon in the second forward-view examination group was hypothesized to be 30%. A minimal sample size of 185 participants per group was required for a significance level of 0.05. The statistical test used in the calculation was the two-sided pooled Z test. At least, 370 participants in total were needed. Therefore, the investigators aimed to recruit a total of 400 participants.

### Outcomes

The primary outcome was to compare the adenoma detection rates of the right colon (ADR of the right colon) between the two groups. The ADR of the right colon was defined as the proportion of patients with at least one adenoma in the right colon. The secondary outcomes were the overall PDR and ADR, PDR of the right colon, per-adenoma miss rate of the right colon, and advanced lesion detection rate. The PDR of the right colon was defined as the proportion of patients with at least one polyp in the right colon. An advanced lesion was defined as a lesion more than 10 mm in diameter, with a villous component on histology or with high-grade dysplasia. The advanced lesion detection rate was defined as the proportion of cases, in which more than one advanced lesion was found. In the SFE group, adenomas/polyps detected on the second examination were defined as missed adenomas/polyps. The per-adenoma miss rate is the number of additional adenomas found on second forward-view examination divided by the total number of adenomas. The numbers of polyps and adenomas per patient were also calculated.

### Statistical analysis

Statistical analysis was carried out by using SPSS version 22.0 (SPSS Inc., Chicago, IL, USA). Continuous variables are reported as the mean and standard deviation (SD), or the median and range, for data with a normal or skewed distribution, respectively. Categorical variables are expressed as percentages. The unpaired Student’s t-test was used to compare normally distributed continuous data; Pearson’s *χ*^2^ test was used to compare categorical variables. A *p* value < 0.05 was considered statistically significant.

## Results

### Baseline demographic and clinical characteristics

**Fig. 1:**
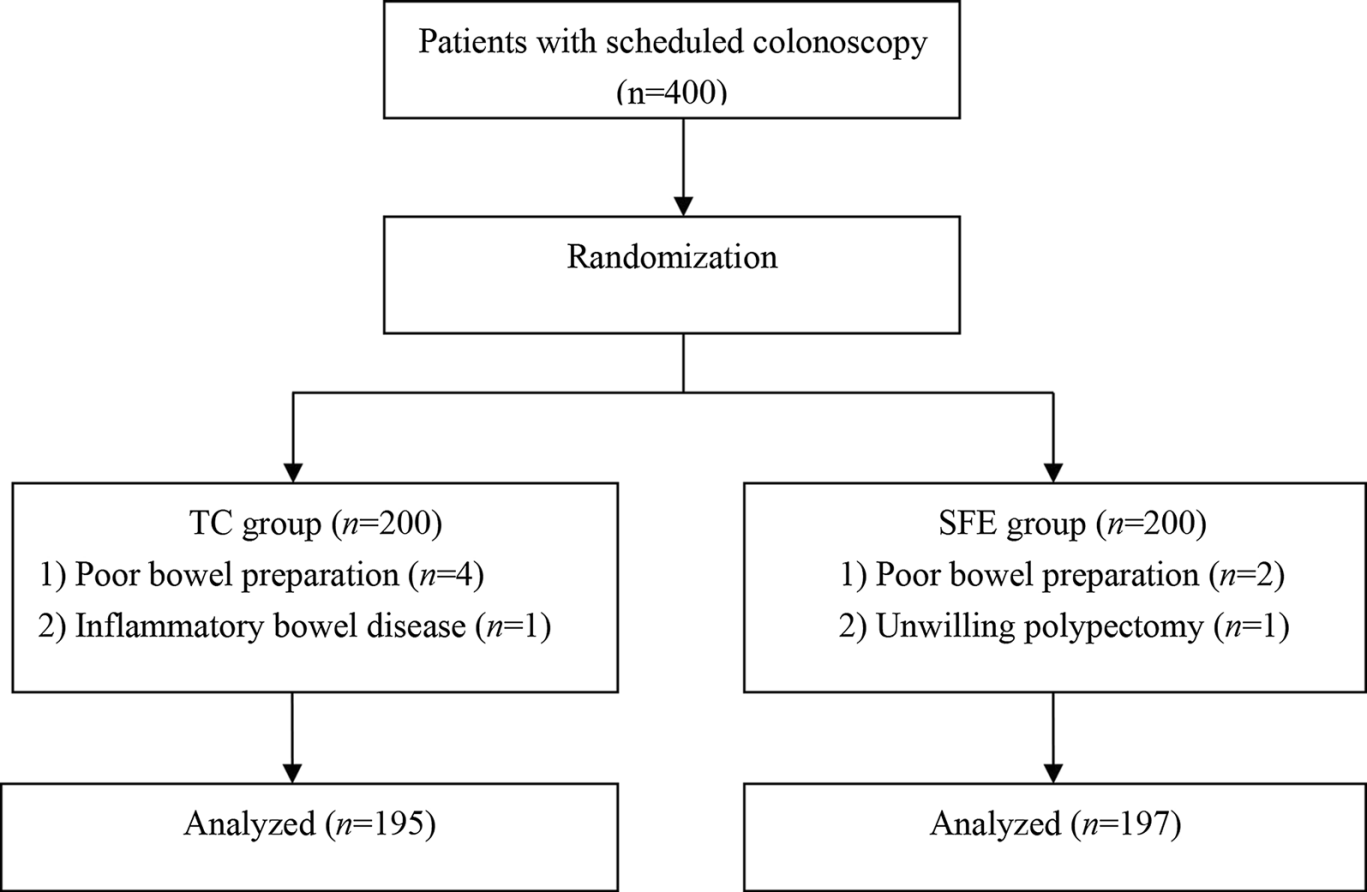
Flow chart for patient inclusions. A total of 400 patients were enrolled and assigned randomly to the TC/SFE group

We recruited 400 patients according to the inclusion criteria and randomly assigned 200 participants to each group. Five patients in the traditional colonoscopy (TC) group and 3 patients in the second forward-view examination (SFE) group were excluded due to inadequate bowel preparation (6 patients), inflammatory bowel disease (1 patient) and unwillingness to undergo polypectomy (1 patient). Finally, 392 patients were included in the analysis, of which 195 patients were allocated to the TC group, while 197 patients were allocated to the SFE group (Fig. 1) . The baseline demographic and clinical characteristics of all included patients are summarized in Table 1.

**Table 1:** Baseline characteristics

Parameter	TC group (*n* = 195)	SFE group (*n* = 197)	*P* value
Age (year)	46.1 ± 13.2	47.7 ± 12.6	0.639
Sex (male/female)	106/89	107/90	0.398
Weight (kg)	62.6 ± 11.4	63.3 ± 11.8	0.337
Height (cm)	165.2 ± 8.0	164.7 ± 7.5	0.494
BMI (kg/m^2^)	22.9 ± 3.1	23.2 ± 3.5	0.450
Indication			0.416
Screening, n (%)	157(80.5)	152(77.2)	
Surveillance, n (%)	38(19.5)	45(22.8)	
Family history of CRC, n (%)	9(4.6)	13(6.6)	0.394
Previous colonoscopy, n (%)	50(25.6)	64(32.5)	0.136
Diabetes, n (%)	9(4.6)	10(5.1)	0.832
Hypertension, n (%)	25(12.8)	32(16.2)	0.336
Smoking, n (%)	50(25.6)	51(25.9)	0.955
Alcohol, n (%)	53(27.2)	52(26.4)	0.814
Intubation time (min)	6.5 ± 4.1	6.8 ± 3.9	0.613
Withdrawal time (min)	6.5 ± 1.3	6.5 ± 1.9	0.100
Second examination time (min)	ND	1.4 ± 0.6	ND
Total duration of colonoscopy (min)	13.0 ± 4.0	14.7 ± 4.8	0.208
Quality of bowel preparation, n			0.884
BBPS 6	40	39	
BBPS 7	54	52	
BBPS 8	65	72	
BBPS 9	36	34	

Data are expressed as the mean ± standard deviation (SD), or percentage

*BMI* body mass index, *BBPS* Boston Bowel Preparation Scale score, *ND* no data, *TC group* traditional colonoscopy, *SFE group* second forward-view examination group

### Polyps and detection rates

For evaluation of the whole colon, a total of 155 and 134 polyps were detected in the SFE group and the TC group, respectively. The mean size of the polyps was 5.26 ± 4.46 mm (range 1–35 mm) in the SFE group and 4.94 ± 2.96 mm (range 2–20 mm) in the TC group (*P* = 0.064). The mean numbers of polyps detected per patient were 0.79 [155/197] and 0.70 [134/195] in the SFE group and the TC group, respectively. The overall PDR did not differ between the two groups (SFE group 41.2% [81/197] vs. TC group 35.9% [70/195], *P* = 0.288). Of the polyps, there were 113 and 99 adenomas in the SFE group and TC group, respectively. The overall ADR did not differ between the two groups (SFE group 32.5% [64/197] vs. TC group 29.7% [58/195], *P* = 0.604). Furthermore, advanced adenomas were found in 15 and 11 patients in the two groups, respectively. The advanced lesion detection rate presented no significant difference between the two groups (SFE group 7.6% [15/197] vs. TC group 5.6% [11/195], *P* = 0.432). The histological characteristics of all colorectal polyps are summarized in Table 2.

**Table 2:** Histological characteristics of colorectal polyps in the study participants

Parameter	TC group (*n* = 134)	SFE group (*n* = 155)	*P* value
Mean size, mm	4.94 ± 2.96	5.26 ± 4.46	0.064
Neoplastic polyps, *n* (%)			0.439
Tubular	95 (70.9)	107 (69.0)	
Tubulovillous or villous	4 (3.0)	3 (1.9)	
SSA	0 (0.0)	2 (1.3)	
Malignant	0 (0.0)	1 (0.6)	
Other polyps, *n* (%)			
Hyperplastic polyps	15 (11.2)	25 (16.1)	
Inflammatory polyps	20 (19.4)	17 (11.0)	

*TC* traditional colonoscopy, *SFE* second forward-view examination, *SSA* sessile serrated adenoma

### Polyp and adenoma detection rates of the right colon

Compared with that in the TC group, a relatively higher proportion of polyps in the right colon was detected in the SFE group (34.2% [53/155] vs. 21.6 [29/134]; *P* = 0.018). The PDR of the right colon showed a significant difference between the SFE group and TC group (17.8% [35/197] vs. 9.7% [19/195]; *P* = 0.021). The mean size of the polyps found in the right colon was 4.92 ± 5.66 mm (range 2–35 mm) in the SFE group and 5.86 ± 4.72 mm (range 2–20 mm) in the TC group (*P* = 0.450) (Table 3). The number of right-sided colon polyps per patient in the SFE group was higher than that in the TC group (0.27[53/197] vs. 0.15[29/195], *P* = 0.003).

**Table 3:** Characteristics of polyps of the right colon

Parameter	TC group (*n* = 29)	SFE group^*^ (*n* = 53)	*P* value
Polyp size, mm	5.86 ± 4.72	4.92 ± 5.66	0.450
< 5 mm	17	36 (13)	0.400
5–9 mm	8	15 (5)	0.945
≥ 10 mm	4	2 (0)	0.096
**Polyp shape, n**			
0-Is	17	27 (12)	0.505
0-Isp	12	23 (6)	0.860
0-Ip	0	3 (0)	0.192

*TC group* traditional colonoscopy, *SFE group* second forward-view examination group

^*^Polyps detected at second exam

**Fig. 2:**
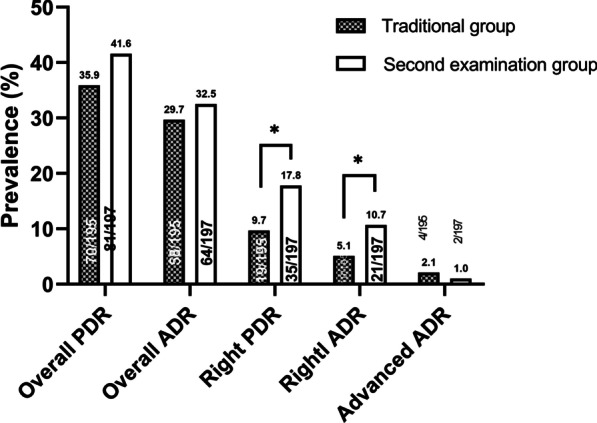
Comparison of ADR and PDR between two groups. PDR, polyp detection rate; ADR, adenoma detection rate. Asterisks indicate *P* value < 0.05 (0.021/0.042 by the χ^2^ test for PDR/ADR of the right colon). P value = 0.245/0.604/0.403 by the χ ^2^ test in overall PDR/ADR and advanced ADR, respectively

In total, 28 adenomas of the right colon were detected in 21 participants in the SFE group, and 14 adenomas in 10 participants in the TC group. The ADR of the right colon was significantly higher in the SFE group than in the TC group (10.7% [21/197] vs. 5.1% [10/195]; *P* = 0.042) (Fig. 2). The advanced lesion detection rate of the right colon was similar in the two groups (SFE group 1.0% [2/197] vs. TC group 2.1% [4/195]; *P* = 0.403).

In the SFE group, a total of 28 adenomas were detected in 21 patients (10.7%). Among these adenomas, 22 were detected on the first forward-view examination and the remaining 6 were detected on the second forward-view examination in 6 patients. None of the six adenomas were advanced adenomas. The per-adenoma miss rate for second forward-view examinations of the right colon was 21.4% (6/28). Miss rates by adenoma size are shown in Table 4.

**Table 4:** Per-adenoma miss rate by adenoma size

	Adenomas found on first forward-view examination	Adenomas found on second forward-view examination	Miss rate* (95% CI)
Numbers	22	6	0.214 (0.052–0.376)
< 5 mm	15	4	0.211 (0.009–0.412)
5–9 mm	5	2	0.286 (−0.166–0.737)
≥ 10 mm	2	0	0.000 (0.000–0.000)

*CI* confidence interval

^*^Per-adenoma miss rate is the number of additional adenomas found on the second forward-view examination divided by the total number of adenomas

### Withdrawal time

There was no statistically significant difference in intubation time or withdrawal time between the SFE group and the TC group (6.5 ± 1.3 min vs. 6.5 ± 1.9 min; *P* = 0.100); however, when the second re-examination time was included, the duration of colonoscopy was 1.7 min longer in the re-examination group than in the TC group, but without statistically significant difference (14.7 ± 4.8 min vs. 13.0 ± 4.0 min; *P* = 0.208).

### Adverse events

No adverse events were observed during the unsedated colonoscopies. Follow-up for post-procedural complications was not performed.

## Discussion

In this single-centre randomized controlled trial, we found that a second forward-view examination of the right colon modestly increase the PDR and ADR to 17.8% and 10.7%, respectively, compared with 9.7% and 5.1% in the traditional colonoscopy group. However, there were no significant differences in the overall PDR, ADR or advanced lesion detection rate between the two groups.

Several previous studies [12, 15, 19, 20] have shown that re-examination of the proximal colon is associated with an increased ADR, as this approach provides a more complete inspection of the colonic mucosa of the proximal colon. Madhav Desai et al. [21] conducted a meta-analysis and found that a second forward view and retroflexed view of the right side of the colon are both associated with improvements in ADR. Our primary outcome is consistent with these findings. Although retroflexion is reported to have a high success rate in terms of maneuverability and a lower risk of adverse events [14–16], endoscopists are more likely to perform forward-view examinations in routine clinical practice. Thus, second forward-view examination might be an optimal choice.

Our study found that there was no significant difference in the overall PDR or ADR between the two groups. This is in line with previous studies [12, 20]. This is because the overall PDR and ADR were already comparatively high in the TC group. Additional detection of polyps and adenomas in the right colon with the second examination made the overall PDR and ADR higher than those in the TC group, but the difference was not statistically significant (overall PDR: 41.6% vs. 35.9%, *P* = 0.245; overall ADR: 32.5 vs. 19.7, *P* = 0.418). Nevertheless, the detection rate of advanced adenoma was higher in the TC group than in the SFE group, but there was no significant difference (*P* = 0.408). It is hypothesized that this observation was due to the small sample of our trial and the fact that large lesions were only detected in 4 and 2 patients in each group. Therefore, more studies with large samples are required to explore this effect.

In the present study, among the polyps detected in the right colon on the second exam, 72.2% were less than 5 mm, and the others were between 5 and 9 mm. When divided by shape, 66.7% were of sessile morphology, and the others were slightly elevated. These characteristics are consistent with those described in previous studies [21, 22]. One explanation is that the colonic mucosa of the right side forms deep and large folds. When small flat lesions are located behind these folds, they can go easily undetected. The second examination inspected the mucosa again carefully. Furthermore, reinsertion may stimulate movement of the mucosa, allowing the endoscopists to view the mucosa from different directions.

In our study, six extra adenomas were found on the second forward-view examination in six patients. A total of 66.7(4/6) of the adenomas were less than 5 mm in diameter, and no advanced adenomas were missed. The miss rate of adenomas in the right colon was 21.4%, which was in line with previous back-to-back studies [22, 23]. Factors influencing adenoma miss rate are variable. The characteristics of the adenomas such as size and shape, withdrawal time and quality of bowel preparation are all closely related to the miss rate [24]. The adenoma miss rate can be reduced with a sufficient observation time during colonoscopy insertion [22]. A second forward-view examination, requiring more observation time, might be such an optimal observation technique for colonoscopists to perform.

Withdrawal time is also considered to be one of the quality indicators of colonoscopy. Compared with that of less than 6 min, a withdrawal time of more than 6 min is associated with a higher ADR [25] and a decreased risk of interval CRC [26]. Thus, a mean withdrawal time > 6 min was guaranteed in the present study, and there was no significant difference between the two groups. When the re-examination time was included, the mean total duration of colonoscopy was 1.7 min longer than that of the traditional colonoscopy group, but the difference was still not statistically significant (14.7 ± 4.8 min vs. 13.0 ± 4.0 min; *P* = 0.208). Therefore, we believe that a second forward-view examination of the right side of the colon is time effective.

Although colonoscopy has been proven to be effective in reducing the risk for CRC, this technique is generally correlated with anxiety, pain, and discomfort among the public. In our study, all colonoscopies were performed without anaesthesia, and none of ceacal intubations failed due to intolerability. In fact, unsedated colonoscopy possesses many advantages and has been preferred over sedated colonoscopy in numerous cancer centres worldwide [27]. Colonoscopists can communicate with participants, leading to good cooperation during the procedure. For instance, patients easily shift their position when required. Patients would also alert colonoscopist when they were in pain. The risk of bowel perforation might be decreased during unsedated colonoscopies [28]. As for ADR, it will not be influenced by sedation. This was observed in a clinical study [29] from Austria that included 52,506 cases of sedated colonoscopies.

The present results provide new evidence that a second forward-view examination could modestly increase the adenoma and polyp detection rate of the right colon during unsedated colonoscopy. Second forward-view examinations are more effective in detecting small adenomas in the right colon. Additionally, the second examination slightly increased the total inspection time of the procedure without statistical significance.

The current study has some limitations. First, the study was a single-centre study, and the sample size was small. A multicentre study with more participants will be required in the future. Second, the endoscopist was not blinded to the protocol. Hence, there was potential psychological effect on attention to lesion detection during the first and second examinations. Perhaps having two different endoscopists perform the first and second examinations could address this problem, but this is not realistic in the real world. Third, the present study did not specifically identify sessile serrated adenomas/polyps (SSAs/Ps). SSAs/Ps is regarded as more likely to become malignant. However, we only found 2 cases among all the participants, but this incidence might be underestimated due to the way we obtain samples. The samples obtained from both snare polypectomy and cold forceps biopsy could not be thoroughly evaluated by pathologists due to the lack of a longitudinal section of the pit on the slides.

## Conclusions

In conclusion, this prospective controlled study revealed that a second forward-view examination of the colon could increase the ADR and PDR of the right colon during unsedated colonoscopies. This simple, safe and time-effective technique might be recommended for routine unsedated colonoscopy.

## Supplementary Information

**Additional file 1: Table S5**. Operators’ ADR.

## Data Availability

The datasets used and/or analysed in the current study are available from the corresponding author on reasonable request.
