# Supplementary material for: Impact of second forward-view examination on adenoma detection rate during unsedated colonoscopy: a randomized controlled trial
Source: BMC Gastroenterol. 2021 May 10;21:213. doi: 10.1186/s12876-021-01783-9 (PMC8111781; doi:10.1186/s12876-021-01783-9)
Supplement: Supplementary file 1 — Additional file 1: Table S5. Operators’ ADR. [file 12876_2021_1783_MOESM1_ESM.pdf]

Table S5. Operators' ADR

Operator	Approximate numbers of colonoscopy experienced annually	numbers of colonoscopy performed	ADR
Keshu Shan	~500	160	29.4%
Hongpeng Lu	~600	173	31.8%
Lei Xu	~800	50	36.0%
Chunjiu Hu*	~800	9	22.2%

ADR, adenoma detection rate.

*Operator Hu quitted the trial after completing 9 cases for personal reason.
